# Chikungunya and Zika Viruses: Co-Circulation and the Interplay between Viral Proteins and Host Factors

**DOI:** 10.3390/pathogens10040448

**Published:** 2021-04-09

**Authors:** Sineewanlaya Wichit, Nuttamonpat Gumpangseth, Rodolphe Hamel, Sakda Yainoy, Siwaret Arikit, Chuchard Punsawad, Dorothée Missé

**Affiliations:** 1 Department of Clinical Microbiology and Applied Technology, Faculty of Medical Technology, Mahidol University, Nakhon Pathom 73170, Thailand; nuttamon.gump@gmail.com (N.G.); sakda.yai@mahidol.ac.th (S.Y.); 2 School of Medicine, Walailak University, Nakhon Si Thammarat 80160, Thailand; chuchard.pu@wu.ac.th; 3 MIVEGEC, Univ. Montpellier, CNRS, IRD, Montpellier, France; rodolphe.hamel@ird.fr (R.H.); dorothee.misse@ird.fr (D.M.); 4 Department of Agronomy, Faculty of Agriculture at Kamphaeng Saen, Kasetsart University Kamphaeng Saen Campus, Nakhon Pathom 73140, Thailand; siwaret.a@ku.th

**Keywords:** Chikungunya virus, Zika virus, antiviral, arbovirus, arbovirus–host interactions

## Abstract

Chikungunya and Zika viruses, both transmitted by mosquito vectors, have globally re-emerged over for the last 60 years and resulted in crucial social and economic concerns. Presently, there is no specific antiviral agent or vaccine against these debilitating viruses. Understanding viral–host interactions is needed to develop targeted therapeutics. However, there is presently limited information in this area. In this review, we start with the updated virology and replication cycle of each virus. Transmission by similar mosquito vectors, frequent co-circulation, and occurrence of co-infection are summarized. Finally, the targeted host proteins/factors used by the viruses are discussed. There is an urgent need to better understand the virus–host interactions that will facilitate antiviral drug development and thus reduce the global burden of infections caused by arboviruses.

## 1. Introduction

Chikungunya virus (CHIKV) and Zika virus (ZIKV) are arboviruses that have globally re-emerged and increased the global burden for public health. The viruses are mainly transmitted by the same mosquito vectors: *Aedes aegypti* and *Ae. albopictus.* However, ZIKV can also be transmitted by blood transfusion and sexual intercourse [1]. Arbovirus infections occur when mosquitoes blood-feed via human skin. During the meals, the mosquito inserts its mouthpiece into the skin and transmits infectious viral particles into both the epidermis and dermis, where inhabitant and migratory cells confront them [2]. People infected by CHIKV usually (up to 90%) develop clinical symptoms characterized by headache, high fever, maculopapular rash, myalgia, arthralgia, and severe asthenia that might last for months or years [3]. In contrast, only 20–25% of ZIKV-infected patients manifest symptoms, usually a “dengue-like” syndrome with a wide range of symptoms (headaches, fever, maculopapular rashes, arthralgia, conjunctivitis, and swelling at the extremities). However, of concern in ZIKV-infected patients are increasing reports of neurological complications (Guillain-Barré syndrome) and neurological birth defects (microcephaly) [4]. Despite the known healthcare threats caused by these viruses, there is still a lack of specific vaccines and therapeutics [5,6]. Normally, patients infected by CHIKV and ZIKV are given anesthetics, anti-inflammatory medications, and supportive care for relieving symptoms. 

Antiviral drugs tend to use one of two main approaches, i.e., direct-acting antivirals (DAAs) and host-targeting antivirals (HTAs). DAAs directly target viral proteins and have proven to be successful. However, DAA compounds often display narrow-spectrum activity, and their development is time-consuming and costly [7]. HTAs are a promising approach that target host factors hijacked by viruses. This strategy demonstrates broad-spectrum activity and is developd using either newly discovered compounds or repurposing existing drugs [8]. Nevertheless, further knowledge of the virology, replication cycles, and host/virus interaction processes of these viruses is needed to help speed antiviral discovery. Therefore, this review article aims to provide updated research information in these areas, with a special focus on CHIKV and ZIKV infections. We hope the review will support further research by identifying new targets for potential therapeutics.

## 2. Virology and Replication Cycle

### 2.1. Chikungunya Virus

**Figure 1:**
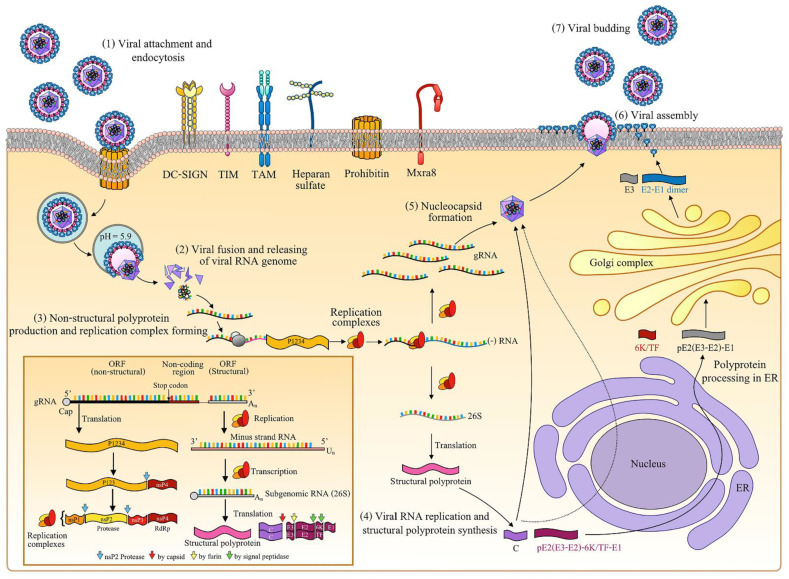
Replication cycle of Chikungunya virus.

The arthropod-borne CHIKV is a member of Togaviridae family and *Alphavirus* genus. There are major four lineages: Asian, Indian Ocean, West African and East, and Central and South African (ECSA) [9]. Its viral particle is 70 nm in diameter and consists of an icosahedral nucleocapsid encapsulated by a lipid envelope. Inside the nucleocapsid, there is a 12-kilobase (kb) positive-sense single-stranded RNA genome. The genomic RNA comprises two open reading frames (ORFs) that are translated into two polyproteins. There is an internal non-coding region, a 5’ untranslated region (UTR) with a type 0 cap, and a 3’ UTR with a poly(A) tail, between the two ORFs (as shown in the lower-left corner of Figure 1) [10,11,12]. The ORF on the 5’ side of the viral genome encodes the non-structural polyprotein that contains non-structural protein 1 (nsP1; 535 aa), nsP2 (798 aa), nsP3 (530 aa), and nsP4 (611 aa). The 3’ ORF produces the structural polyprotein that is further processed into capsid (C) (261 aa), envelop (E) 3 (64 aa), E2 (423 aa), 6K/TF (61 aa), and E1 (439 aa) proteins [11,13,14].

The structural proteins of CHIKV are involved in the viral entry, assembly, and budding steps of the viral replication cycle. The structural C protein, comprised of two distinct domains (protease domain (C-terminal) and RNA binding domain (N-terminal) [15]), is used to form an icosahedral nucleocapsid that is covered by the envelope. Moreover, the CHIKV C protein contains the nuclear localization and exportation signals (NLS and NES, respectively), which are associated with protein transportation between nucleus and cytoplasm, a process which may be important for viral replication [16]. The CHIKV envelop protein is a host-derived plasma membrane with trimeric spikes, a trimer of glycoprotein E2, and an E1 heterodimer [17]. Both E2 and E1 predominantly contain β-sheet secondary structures, and each have three domains, which are the C, A, and B domains in E2 and the III, I, and II domains in E1 [10]. While the function of E1 glycoproteins is to mediate the virus–host cell membrane fusion, E2 facilitates the binding of virus and host–cell attachment factors [18]. The heterodimer of E2 and E1 is stabilized by the E3 protein, and it involves the translocation of viral structural polyproteins to the endoplasmic reticulum (ER). E2 and E3 are produced by the cleavage of the E2 precursor (pE2) by furin protease [10,17,19], a small hydrophobic protein 6 K/TF that is involved in membrane permeabilization and viral budding [20,21].

The CHIKV non-structural protein (nsP1) expresses enzymatic activities (guanine-N7-methyltransferase and guanylyltransferase) for 5’ capping at the end of viral genome, a function necessary for viral RNA translation and the prevention of RNA degradation [22]. In addition, nsP1 helps to promote virion release by downregulating the expression of the virus restriction factor, tetherin [23]. On the nsP2 protein, the C-terminal contains a cysteine protease needed for non-structural polyprotein processing [24,25], while the N-terminal is composed of a helicase and a nucleoside triphosphatase (NTPase) necessary for RNA unwinding and viral RNA capping [26,27]. 

nsP3 is comprised of a macrodomain, an alphavirus unique domain (AUD), and a hypervariable domain (HVD) associated with a viral replication complex that promotes RNA replication and virulence [28,29,30]. The nsP4 functions as an RNA-dependent RNA polymerase (RdRp) essential for CHIKV RNA synthesis [31,32]. The non-structural polyprotein is first produced as nsP1234. Then, the polyprotein is cleaved into nsP123 and nsP4 by its protease, followed by further cleaving with the same protease to produce nsP1, nsP2, and nsP3. Finally, nsP1, nsP2, nsP3, and nsP4 form a “replication complex” that is responsible for viral RNA synthesis (negative-stranded, positive-stranded, genomic, and sub-genomic) [25,33].

The replication cycle of CHIKV is shown in Figure 1. CHIKV enters the host cell via clathrin-mediated endocytosis through the binding of its E2 protein with host attachment factors on the cell surface [34,35]. However, clathrin-independent endocytosis also has been reported [36]. The mammalian-cell attachment factors responsible for CHIKV binding include glycosaminoglycans (GAGs) (heparan sulfate, dextran sulfate, chondroitin sulfate, and dermatan sulfate [18]), dendritic-cell-specific ICAM-grabbing non-integrin (DC-SIGN) [37], TIM receptor family (T cell immunoglobulin mucin (TIM)-1), TAM (tyrosine protein kinase receptor 3 (Tyro3), Axl, and Mer) receptor family [38,39], prohibitin [40], and matrix remodeling-associated protein 8 (Mxra8) [41]. After internalization, the acidic environment in the endosome drives the fusion of the host cell endosomal membrane and viral E1 protein, resulting in the release of nucleocapsid and the subsequent release of the viral RNA genome into the cytoplasm [42]. 

The positive-sense single-stranded RNA genome is then translated into nsP1234 and further processed to form a viral replication complex. The complex then synthesizes viral negative-sense RNA, which serves as a template for positive-strand genomic (49S) and sub-genomic (26S) RNA synthesis [25,33]. The 26S sub-genomic RNA is translated into structural polyproteins (C-pE2(E3-E2)-6K/TF-E1). Later, the C protein is released through the cleavage of its autoprotease in either the cytoplasm or nucleus, and the remaining pE2(E3-E2)-6K/TF-E1 polypeptide traffics to the ER for further processing [43]. Polypeptide cleaving occurs in the ER by host signal peptidase to form pE2(E3-E2), 6K/TF, and E1. The pE2(E3-E2) and E1 form a heterodimer before migrating to the Golgi complex. Furin protease in the Golgi complex drives the generation of mature E2 and E3, which subsequently dissociate to form an E2-E1 heterodimer during export from the Golgi complex to the plasma membrane [10,17,19]. The E2-E1 heterodimer arrives and embeds in the plasma membrane [17]. Finally, the nucleocapsid (built from oligomerization of the C protein covering viral genomic RNA) transports to host cell plasma membrane, where the nucleocapsid acquires an envelope before release as an assembled virus particle (mature virion) from the infected cell via the budding mechanism [44].

### 2.2. Zika Virus

The arbovirus, ZIKV, another global threat, is a member of the family Flaviviridae in the *Flavivirus* genus, which includes other human pathogenic viruses such as dengue virus (DENV), Japanese encephalitis virus (JEV), and West Nile virus (WNV) [45]. Two major lineages of ZIKV have been identified: African and Asian [46]. The mature virion is approximately 50 nm in diameter [47] and, as with other *Flaviviruses*, its structure is composed of an outer envelope enclosing an inner icosahedral nucleocapsid that contains a 10.8-kb positive single-stranded RNA [48]. Distinct from CHIKV, the ZIKV genome consists of a single ORF flanked by 3’ and 5’ UTRs, and the end of the 5’ UTR contains a viral promoter and a type I cap [49,50,51]. The ORF encodes a large single polyprotein that is processed into three structural proteins, C, pre-membrane (prM), and E, as well as the seven non-structural proteins NS1, NS2A, NS2B, NS3, NS4A, NS4B, and NS5 [50,52,53,54].

ZIKV structural proteins play an important role in the formation of infectious virions, receptor binding, viral assembly, and release. The envelope protein, which is prominent on the surface of virus particles, consists of four domains: a stem–transmembrane domain pair involved in membrane attachment and three β-sheet domains designated as ectodomains I, II, and III. Domain II is required for viral fusion, whereas domain III is needed for receptor binding [55,56]. Situated under the E protein, there is a membrane (M) protein, a part of the prM protein processed by the host furin protease during virion maturation and adherent to the virion’s host-derived lipid membrane. Inside the lipid membrane, icosahedral nucleocapsid constructed from structural C protein is encapsulated [47,48]. The viral C protein is formed as a homodimer with two different parts on opposite sides: a hydrophobic side thought to bind with the viral membrane protein and a highly positively charged side theorized to contact with the viral RNA genome. Together, these suggest that C protein strongly affects the viral assembly process [57].

ZIKV non-structural proteins are implicated in viral genome replication, particle assembly, and host immune evasion. The NS1 protein, a membrane-associated homodimer form, plays an important role in viral replication by forming a replication complex. Moreover, the NS1 protein is secreted from the infected cell as hexameric proteoliposomes that aid immune evasion through the alteration of the complement system [58,59,60] and reducing the type-I interferon (IFN) production at the TANK binding kinase 1 (TBK1) phosphorylation step [61]. ZIKV NS1 protein’s structure is similar to that of NS1 of other flaviviruses. Therefore, it is difficult to differentiate this virus from other members of the family via NS1. 

However, the ZIKV NS1 protein shows unique electrostatic potentials on bound host factors and protective antibodies [62]. NS2A, NS2B, NS4A, and NS4B are membrane-associated hydrophobic proteins located within ER membranes and are implicated in the viral replication complex. Moreover, the NS2A protein interacts with viral RNA and proteins to facilitate virion assembly [63,64]. It also acts as an innate immune response antagonist by blocking TBK1 phosphorylation and suppressing type-I IFN [61]. NS2B is a cofactor with NS3 to form a serine protease complex that is involved in viral polyprotein processing [65]. The ZIKV NS3 protein has two functional domains—protease and helicase—that exhibit multiple enzymatic activities such as serine protease [65], RNA helicase [66], and NTPase [67]. Therefore, it plays multiple roles in the virus replication cycle, e.g., the unwinding of double-stranded RNA during virus replication and polyprotein processing [66]. For NS4B, in addition to associating with the replication complex during viral replication, it also acts in immune evasion by blocking interferon signaling by targeting to TBK1 [61]. The NS5 protein also suppresses IFN signaling through the proteasomal degradation of human STAT2 [68]. The ZIKV NS5 protein has two domains—the N-terminal methyltransferase (MTase) and C-terminal RNA-dependent RNA polymerase (RdRp) domains—that are essential for viral genome synthesis, genome stabilization, and, as mentioned earlier, host immune response evasion [69,70].

**Figure 2:**
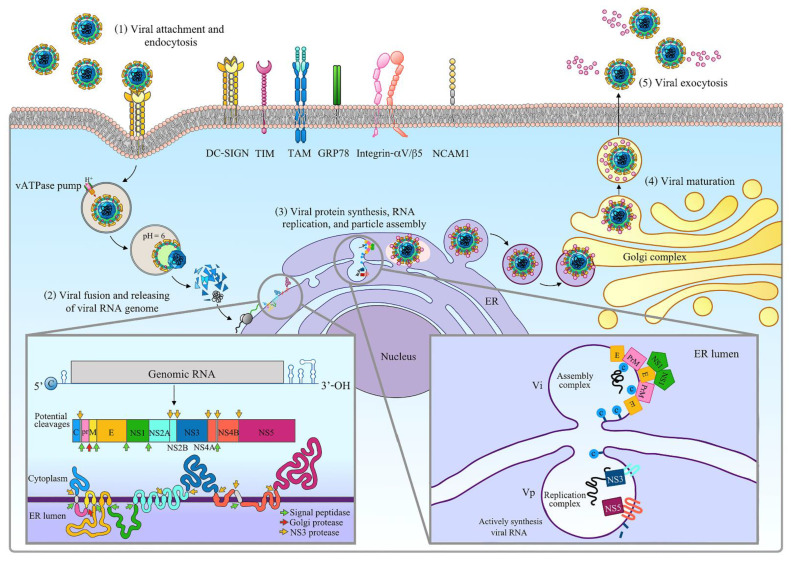
Replication cycle of Zika virus.

As shown in Figure 2, the ZIKV replicative cycle begins with the attachment of the viral E proteins to specific attachment factors on the host cell surface. Several factors are responsible for ZIKV entry including DC-SIGN, TIM receptor family (TIM-1 and TIM-4), TAM receptor family (AXL and Tyro3) [71], and glucose-regulating protein 78 (GRP78) [56]. 

Recently, two attachment factors have been reported to mediate ZIKV infection: the integrin-αV/β5, which facilitates viral infection of neuronal stem cells [72], and the neural cell adhesion molecule (NCAM1), which facilitates the infection of human astrocytoma cells [73]. After these interactions, the virus subsequently internalizes via clathrin-dependent or clathrin-independent endocytosis [74,75]. Then, a vacuolar ATPase pumps protons into the endosome to create an acidic environment that triggers fusion between viral envelope proteins and endosomal membranes. This is followed by the C protein degradation of the viral nucleocapsid, resulting in the release of viral RNA into the cell cytoplasm [75]. In host cell cytosol, viral RNA translation occurs immediately after release and results in the generation of a polyprotein with an N-terminal ER localization signal. This polyprotein traffics to localize at the ER membrane, which comprises prM, E, NS1 and parts of NS2A, NS4A, and NS4B located inside the ER lumen, while C, NS3, and NS5 face toward the cytoplasm. This polyprotein is cleaved by host proteases (cleaving ER lumen-facing parts) and a viral protease (cleaving cytosol-facing parts) to obtain virus structural and non-structural proteins [76]. Then, the replication complex forms and viral genome synthesis occurs. NS1, NS2A, NS2B, NS4A, and NS4B transmembrane proteins associate with modified lipids that provide a molecular scaffold for complex replication. The complex then induces ER membrane rearrangements that facilitate the generation of vesicle pockets (VPs) where the replication complex is located [77,78,79].

Viral RNA replication occurs through the replication complex in the VPs. The positive single-stranded RNA is transcribed using NS5 proteins (RdRp) to produce dsRNA which is comprised of both positive and negative strands. This dsRNA is unwound by the NS3 helicase to generate both positive and negative ssRNAs. The negative ssRNAs serve as templates for further transcription, while positive ssRNAs combine with prM, C, and E proteins in the ER to assemble immature virions. The immature particles subsequently traffic from ER to Golgi complex and the trans-Golgi network where the acidic environment reveals the prM cleavage site. Finally, the prM is cleaved by a host furin protease, thus allowing for particle maturation, followed by the release of mature virions from infected cells by exocytosis [80,81,82].

## 3. Co-Circulation of Chikungunya and Zika Viruses 

### 3.1. Geographic Distribution of Chikungunya and Zika Viruses

**Figure 3:**
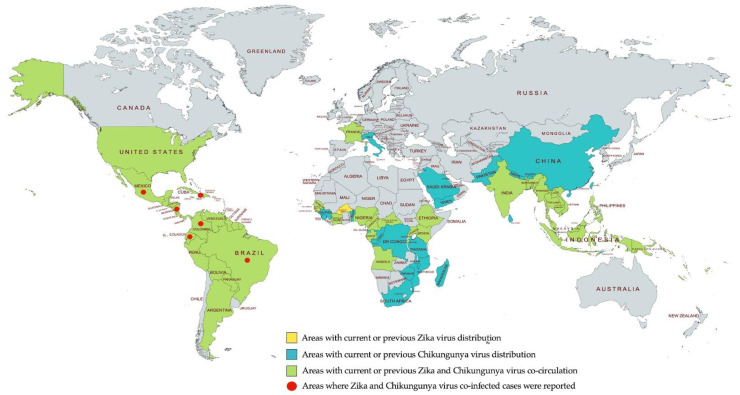
Geographic distribution of Chikungunya and Zika viruses. Information retrieved from the U.S. CDC website, last accessed in February 2021 [126,127]. Map was created with mapchart.net.

CHIKV was first reported in southern Tanzania in 1952 [83], followed by occasional reports of CHIKV-infected cases in Africa [84,85,86] and Asia [87,88,89,90]. The first epidemic in Asia was documented in Bangkok, Thailand, in 1958 [88]. Since then, CHIKV has rapidly spread worldwide. An outbreak in Kenya in 2004, which subsequently caused infections in most islands of the Indian Ocean over the next two years [91,92,93,94]. CHIKV from the Indian Ocean spread to many countries in Asia such as India [95,96], Sri Lanka, Myanmar [97], and Thailand [98], as well as to northern Italy in 2007 (the first report in Europe) [99]. In 2010, the first autochthonous case was reported in Europe [100], and, in the same year, CHIKV was also reported in several countries in Asia [101,102,103]. In the Americas, the first autochthonous case was identified in Saint Martin in 2013 [104]. CHIKV subsequently caused viral infections through the North, Central, and South Americas [105,106]. As of 2021, 115 countries/territories have been affected by CHIKV (Figure 3). 

ZIKV was first identified in 1947 from a sentinel rhesus monkey during a yellow fever project in the Zika forest near Entebbe in Uganda [107]. The first human cases were reported in 1952 from Uganda and the United Republic of Tanzania [108]. Since then, ZIKV-infected cases have occurred sporadically in Africa [109,110,111,112,113,114] and Asia [90,115,116,117,118]. A large outbreak of ZIKV occurred outside Africa and Asia in 2007 in the Pacific Ocean country of the Federated States of Micronesia (on “Yap Island”), thus causing infection of approximately 73% of residents [119]. 

In 2013–2014, ZIKV caused an outbreak in French Polynesia and was subsequently transmitted to other Pacific islands including Easter Island, New Caledonia, and the Cook Islands [120,121,122,123]. A year later, in 2015, the first outbreak of ZIKV in the Americas took place in Brazil and rapidly spread through many countries in the region [124,125]. As of 2021, 86 countries/territories worldwide report having ZIKV-infected cases (Figure 3). CHIKV and ZIKV co-circulate in more than 80 of these countries/territories (Figure 3). 

### 3.2. Cases of Chikungunya and Zika Virus Co-Infection

CHIKV and ZIKV are arboviruses, meaning that they can be transmitted by blood-feeding arthropods. For ZIKV, the main vector for transmission from human to human is *Aedes* mosquitoes [128,129,130,131]. CHIKV is mainly transmitted by *Ae. aegypti.* In 2006, a CHIKV E1 mutation (A226V) resulted in increased viral replication in *Ae. albopictus,* and this mosquito species became another important vector of the virus [132,133,134,135]. As CHIKV and ZIKV co-circulate in multiple countries worldwide, there is a high likelihood of co-infection in a patient. Indeed, 142 cases of CHIKV and ZIKV co-infection have been reported from six countries (a country in Central America, two countries in North America, and three countries in South America).

#### 3.2.1. Haiti 

From May 2014 to February 2015, during the CHIKV outbreak in the Americas, Jacob and team investigated schoolchildren with febrile illness to determine diagnosis, clinical characterization, and epidemiological features. In the same period, DENV and ZIKV were reported in the area [125,136]. Therefore, blood samples from children were tested for these viruses and identified six co-infections of CHIKV and ZIKV by quantitative reverse transcription polymerase chain reaction (qRT-PCR) [137]. 

#### 3.2.2. Colombia

DENV has been endemic in Colombia for a long time [138], while CHIKV arrived in 2014 [139,140] and was followed by ZIKV in 2015 [141], thus leading to the local co-circulation of these viruses. Between August 2015 and April 2016, blood samples obtained from patients with febrile illness at the Jorge Cristo Sahium Hospital were found positive for DENV, CHIKV, and ZIKV by conventional PCR, qRT-PCR, and genotypic sequencing. Eight patient samples were positive for both CHIKV and ZIKV, while three samples were positive for DENV, CHIKV, and ZIKV, indicating co-circulation and co-infection by the viruses in Colombia [142]. From the end of 2015 to December 2016, ZIKV-suspected samples from the National Surveillance System in Public Health (SIVIGLIA) were investigated by singleplex and multiplex qRT-PCR for DENV, CHIKV, and ZIKV. Twenty-eight CHIKV and ZIKV co-infected samples were found, and five of these co-infected cases were fatal [143]. 

In addition to these two cross-sectional studies, there were reports of autochthonous cases in 2016. A study reported co-infection in a 49-year-old male who traveled through the Sucre and Bolivar departments of Colombia. His blood sample was reported positive for DENV and ZIKV RNA genomes, as well as for CHIKV IgM antibodies [144]. In the same year and department, a 33-year-old pregnant woman was determined to be positive for DENV serotype 2, the CHIKV Asian genotype, and ZIKV Asian lineage by nested RT-PCR and sequencing, indicating co-infection with DENV, CHIKV, and ZIKV [145]. In 2018, a pregnant woman from the southern part of Colombia was found to have a co-infection of *Toxoplasma gondii*, CHIKV, and ZIKV using PCR assays of amniotic fluid. In this case, the pregnancy was medically terminated at 29 weeks of gestation [146]. 

Another 2016 report describes a 40-year-old woman living in Florida (no local transmission of CHIKV or DENV at that time) who traveled to Colombia for seven days. She became ill and was studied with tests that included qRT-PCR for arbovirus—she was found positive for ZIKV. Arthralgias persisted in the patient for months, and her sample was further evaluated by inoculating it into cell cultures. Using qRT-PCR, CHIKV and ZIKV were observed in the cultures, thus indicating co-infection by CHIKV and ZIKV in this patient. Viral genome sequencing was also performed, and the results showed a close relationship to the CHIKV and ZIKV strains previously isolated in Colombia [147]. This case was investigated in the United States after her return, and it warned of a high risk for CHIKV and ZIKV introduction into a new region of the world since transmission would be easy via one bite of a vector mosquito. 

#### 3.2.3. Brazil

After CHIKV was introduced into the Americas in 2013, it was reported to cause the first outbreak in Brazil in 2014 [148], while ZIKV was first reported in Brazil in 2015 [124,125], suggesting the co-circulation of CHIKV and ZIKV in that country. From May 2015 to 2016, Brazilian patients with illness suspected of arbovirus infection were investigated for CHIKV and ZIKV using qRT-PCR. The result showed two cases of co-infection with CHIKV and ZIKV [149]. These data indicated co-infection during virus co-circulation in Brazil, even in a decreasing period of ZIKV infection in which asymptomatic patients might still spread ZIKV. Therefore, detecting and monitoring co-infected cases is still important. In addition, during this co-circulation period, there were many reports of co-infected cases in Brazil. One study reported two cases of co-infection with CHIKV and ZIKV from the ECSA lineage of CHIKV and the Asia lineage of ZIKV [150]. Another study investigated samples from the University Hospital Clementino Fraga Filho/Federal University of Rio de Janeiro with qRT-PCR and reported one case of the CHIKV and ZIKV co-infection [151]. Because a ZIKV infection during the gestation period can cause central nervous system malformations in the fetus, pregnant women were studied from September 2015 through May 2016. Three co-infections of CHIKV and ZIKV in these women at the acute febrile illness clinic were detected by IgM antibodies and viral RNA [152]. 

During the same period, a study focused on differential diagnosis in patients with neurological disorders. A sample of patients who had developed a neurological complication associated with ZIKV was studied. Five cases of CHIKV and ZIKV, as well as one case of a DENV, CHIKV, and ZIKV co-infection, were detected using ELISA and qRT-PCR [153]. From March to May 2016, an investigation of samples from private hospitals using qRT-PCR found 36 cases of co-infection by CHIKV and ZIKV. CHIKV-positive samples in this study were randomly chosen for further analysis by NGS and revealed the ECSA lineage of CHIKV [154]. 

In 2016, a 74-year-old male resident from Recife, northeast Brazil, was reported to have a co-infection by CHIKV and ZIKV with severe meningoencephalitis. The CSF and serum of the patient were tested for ZIKV and CHIKV using qRT-PCR and IgM serology, and they found ZIKV and CHIKV RNA in serum and IgM against CHIKV in CSF. However, there were no antibodies against ZIKV in either the CSF or serum [155]. Moreover, the first fatal pregnancy, a 20-year-old at 21 weeks of gestation, that was associated with CHIKV and ZIKV co-infection was reported. Her blood sample was positive for IgM and IgG specific for DENV and CHIKV, and it was IgG specific for ZIKV. Additionally, CHIKV RNA was detected by qRT-PCR. CHIKV and ZIKV RNA were detected in the placenta and kidney, respectively, of the dead fetus, associating the fetal death with the co-infection [156]. Recently, from the end of 2017 to early 2018, samples from several cities in Minas Gerais were tested by ELISA for antibodies against DENV, CHIKV, and ZIKV. The results showed five cases that were positive for IgM specific to CHIKV and ZIKV, and one case positive for IgM specific to all three viruses [157].

#### 3.2.4. Nicaragua

The first CHIKV autochthonous case in Nicaragua was recorded in September 2014, whereas the first ZIKV case was identified in January 2016 [125,158,159]. During the epidemic of these two viruses, ten co-infected cases with CHIKV and ZIKV and four co-infected cases of DENV, CHIKV, and ZIKV were detected using a ZDC assay (a multiplex qRT-PCR for detecting ZIKV, DENV and CHIKV) [159]. Furthermore, between September 2015 and April 2016, serum samples collected from Ministry of Health facilities and tested using the same assay detected sixteen cases of CHIKV and ZIKV and six cases of DENV, CHIKV, and ZIKV co-infection [160]. 

#### 3.2.5. Ecuador

DENV has been endemic in Ecuador since 1988 [161], while CHIKV was first identified in 2014 and continues to be epidemic [162]. ZIKV was introduced in January 2016 [163], and its outbreak might have driven the co-circulation and co-infection that year. A report of co-infection at the Hospital Luis Vernaza in Guayaquil was published with three cases that were diagnosed in early 2016. The first patient was a 43-year-old male, the second was a 43-year-old woman who lived in the United States and traveled to Ecuador for two weeks, and the last was a 57-year-old female with severe neurologic symptoms. All patients were found to be positive for DENV, CHIKV, and ZIKV RNA using a ZDC assay [164]. Furthermore, a total of four CHIKV and ZIKV co-infection cases and four DENV, CHIKV, and ZIKV co-infection cases were detected by a ZDC assay on CSF samples from the Hospital Luis Vernaza in Guayaquil in 2016 [165].

#### 3.2.6. Mexico

In Mexico, dengue virus re-emerged in 1978 after being absent since 1961 [166], while the first cases of CHIKV and ZIKV were reported in 2014 and 2015, respectively [167,168]. These three arboviruses are considered endemic in this country and co-circulate in the same areas. However, dengue-infected cases were detected in 2018, and outbreaks occurred in several states in 2019. CHIKV and ZIKV seemed to decrease in circulation these two years, but they were still present [125,136,169]. 

During a DENV outbreak February to August 2019, blood samples from pregnant women who lived in the central region of the state of Chiapas were collected and found positive for DENV, CHIKV, and ZIKV using qRT-PCR. Cases of co-infection with CHIKV–ZIKV and DENV–CHIKV–ZIKV were found [170].

## 4. Interplay of Virus Proteins and Host Factors

To develop antiviral agents, a greater understanding of virus–host interaction is required. Summaries of the viral protein–host factor interactions of CHIKV and ZIKV are shown in Table 1 and Table 2, respectively. 

### 4.1. Cellular Entry and Targets

**Figure 4:**
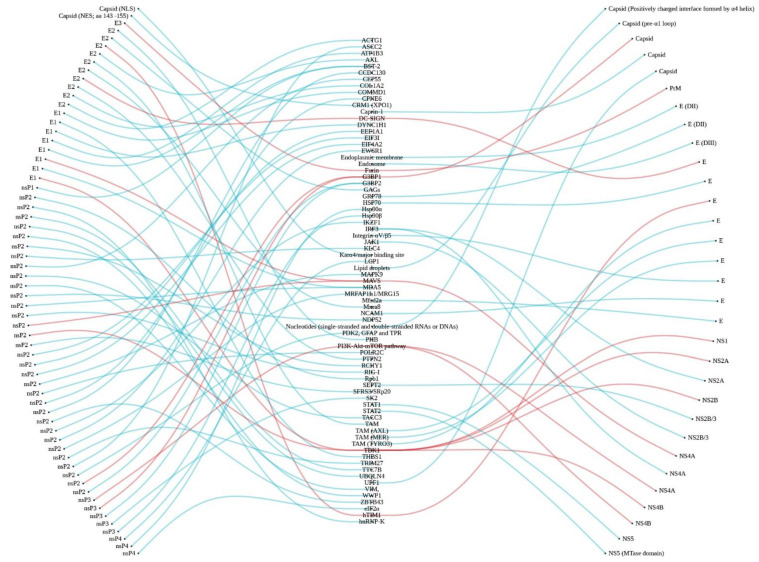
Eye diagram linking host factors (**middle**) with Chikungunya virus proteins (**left**) and Zika virus proteins (**right**). Red lines represent human proteins that can interact with both Chikungunya and Zika virus proteins.

CHIKV and ZIKV are enveloped arboviruses which come from different families. However, these viruses display similar components: envelope, membrane, and icosahedral nucleocapsid around a viral RNA genome. Binding to and entering human host cells begin with binding between viral proteins and host cell attachment factors. The envelope protein E2 of CHIKV and E protein of ZIKV have been documented to be the viral proteins involved in this task [18,71]. Interestingly, among the host cell attachment factors, three identical factors have been reported: DC-SIGN, hTIM-1, and TAM [37,38,39,71] (Figure 4). 

DC-SIGN is a pattern recognition receptor predominantly expressed on the surface of dendritic cells and macrophages [171]. It serves as an attachment factor for various types of viruses, including alphavirus (CHIKV [37]), flavivirus (DENV [172], JEV [173], HCV [174], WNV [175], and ZIKV [37]), filovirus (EBOV [176]), herpesvirus (CMV/HHV-5 [175], and Kaposi’s sarcoma herpes virus [177]), orthomyxovirus (influenza virus [178]), retrovirus (human immunodeficiency virus 1 (HIV-1) and HIV-2 [179,180]), and coronavirus (human coronavirus 229E [181], SARS-CoV and SARS-CoV-2 [175,182,183,184]). Polymorphism in DC-SIGN was reported as a risk factor for CHIKV infection and pathology [185]. This phenomenon is based on an alteration of the CD209 promoter gene (A/G (rs4804803) at position 336) and the greater frequency of this genotype in Chikungunya-infected patients compared to healthy controls [171,186]. Peer-reviewed publications have reported that DC-SIGN is important for the attachment and internalization steps of both alphaviruses and flaviviruses [187,188,189,190]. Interference with DC-SIGN activity can inhibit virus infection and is thus a potential target for novel antiviral therapies. In 2014, Varga and his team synthesized multivalent Man glycodendrimers to compete with virus E protein for DC-SIGN interaction [191]. 

Among multivalents, the hexavalent-glycodendrimer has demonstrated the highest inhibition activity for HIV and DENV2 infection in B-THP-1 and Raji cells with DC-SIGN-overexpression, respectively. This information could inform the discovery of specific molecules that can compete with the binding of viral E protein to DC-SIGN on host cells. In contrast to mimicking viral E protein production, inhibitors directly targeting viral E proteins were discovered. Three plant lectins (*Galanthus nivalis* agglutinin (GNA), *Hippeastrum* hybrid agglutinin (HHA), and *Urtica dioica* (UDA) isolated from the amaryllis) were reported as DENV envelope protein binding agents that were subsequently found to prevent virus–DC-SIGN attachment [192]. Recently, Prado Acosta and team announced that DC-SIGN-overexpressed cells show significantly enhanced CHIKV and ZIKV infections, confirming DC-SIGN’s function as a virus attachment factor. There was a report that the interaction between the surface layer (S-layer) proteins of *Lactobacillus acidophilus* (a bacteria in the human intestine) and DC-SIGN markedly inhibits H9N2 and Junin viral infections [193,194]. A further investigation of the S-layer’s inhibition ability found that blocking DC-SIGN by pretreating cells with the S-layer significantly decreases CHIKV and ZIKV infectivity in a dose-dependent manner [37]. However, further study is needed to clarify the inhibitory mechanisms of the S-layer.

hTIM-1 (human T-cell immunoglobulin and mucin 1) is a human TIM protein from a family of phosphatidylserine (PS) receptors that are mainly expressed in T helper (Th) 2 cells, kidney epithelia, and a broad range of mucosal epithelia including the trachea, conjunctiva, and cornea [221,222]. hTIM-1 is reported to be a receptor for attachment by various enveloped viruses [39]. Kondratowicz and team documented that TIM-1 acts as an EBOV receptor both in vitro and in vivo. The overexpression of TIM-1 in restricted cell lines significantly enhances EBOV infection, while, in contrast, TIM-1-silenced cells markedly decrease the viral infection of even highly permissive cells [221,222]. In keeping with in vitro results, in vivo experiments with TIM-1-sufficient or -deficient BALB/c mice showed that TIM-1 increases viral load and the associated mortality [223]. These results imply that TIM-1 is essential for, at least, viral entry and pathogenesis. Recently, Ichimura et al. demonstrated that kidney injury molecule-1/T cell immunoglobulin mucin domain 1 (KIM-1/TIM-1) is a novel receptor for SARS-CoV-2. Virus binding can be blocked by anti-KIM-1 antibody and TW-37, a discovered inhibitor of KIM-1/TIM-1, mediated endocytosis [224]. This result supported the crucial role of TIM-1 as a virus receptor. The finding of TIM-1 inhibitor(s) may provide an additional modality to prevent or treat COVID-19.

In CHIKV, TIM-1 was identified as an attachment factor that promotes viral infection rather than being a specific receptor. In TIM-1-overexpressed cells, CHIKV infection is moderately enhanced and the transfection of CHIKV-pseudotype viral vectors is slightly inhibited by PS liposomes [38]. Similarly, ZIKV infection is partially decreased by neutralizing Ab specific for TIM-1 and by an RNA silencing technique to downregulate TIM-1 before virus infection [71]. However, the downregulation of TIM-1 together with AXL expression in A549 cells totally abrogated ZIKV infection. These data suggested that TIM-1 is a co-factor for the viral attachment and entry of CHIKV and ZIKV. Though TIM-1 was reported to be a virus receptor or attachment factor for a long time, data regarding its inhibitors are limited. Li and team found that HIV-1 Nef proteins and lentivirus accessory proteins antagonize TIM-mediated cell restriction, while, SERINC5 host cell restriction factors stabilized the expression of TIM-1 [225]. 

However, the mechanisms of the Nef inhibition of TIM-1 need to be further investigated. Therefore, the investigation of TIM-1 and its inhibitors impact upon virus interactions may help to develop effective antivirals that could reduce viral load and severity during virus infection. 

The TAM receptor family consists of transmembrane receptor protein tyrosine kinases (PTKs) that regulate PTK activity within their cytoplasmic domains. The name “TAM” derives from the first letter of its three members: Tyro3, Axl, and Mer [226]. Axl is reported to enhance EBOV and MARV in the entry step [227]. Likewise, Axl also promotes the CHIKV infection of hAxl-293T-overexpressed cells. The proposed mechanism is that Axl binds and internalizes various viruses through PS-binding bridging proteins (Gas6 and/or Protein S) in serum [39]. 

Axl is highly expressed in several types of ZIKV-permissive cells. In in vitro experiments, Axl acts as a virus attachment factor or as a signaling receptor to enhance virus infection [71,211,213,228,229,230,231,232,233,234]. In contrast, in vivo experiments have shown that the ZIKV infection of some mice organs (eyes, brain, and testes) is independent of TAM receptors [209,235]. Furthermore, the loss of Axl does not impact ZIKV infectivity, as shown in experiments deleting Axl, using CRISPR-Cas9 technology, in human neural progenitor cells and cerebral organoids [236]. Collectively, the data (though inconclusive) presented here suggest that: (1) mice may have entry receptors/attachment factors that are not expressed in human cells or (2) human cells do not recapitulate the expression of a wide range of virus entry receptors/attachment factors. Finding the relevant entry receptors/attachment factors will be essential for a more complete understanding of ZIKV virology and cell tropism. 

Furthermore, Wang et al. recently reported that Axl is a candidate receptor of SARS-CoV-2. They used both pseudotype and wild-type SARS-CoV-2, and they found that Axl-HEK293T-overexpressed cells have enhanced virus infection while Axl-H1299-depleted cells had markedly reduced viral infection [237]. This emphasized the significance of Axl in a wide range of virus receptors in humans. These data implied that TIM and TAM, PS-binding proteins, promote infection by diverse families of enveloped viruses and are, therefore, candidate targets for broad-spectrum antiviral therapies.

### 4.2. Host Cell Immune Responses and Viral Immune Evasion Mechanisms

Initially, upon virus infection, host innate immune systems sense single or double-stranded (ss or ds) RNAs in infected cells by pathogen-associated molecular pattern (PAMP) recognition. For example, retinoic acid-inducible gene-I (RIG-I) recognizes short 5′-triphosphorylated ssRNA and short dsRNA, and melanoma differentiation-associated protein 5 (MDA5) participates in the recognition of longer dsRNA products [238,239,240]. Using a PCR array and qRT-PCR, our team reported that *IFIH1* (MDA5) was upregulated during wild-type CHIKV infection in human skin fibroblasts and that the expression level was downregulated when infected in the presence of mosquito saliva [241]. Using CHIKV proteins cloned into an expression vector, nsP2, E1, and E2 were found to strongly downregulate MDA5, while nsP2 and E1 (but not E2) inhibited RIG-I [242]. This information implicates MDA5 and RIG-I as major sensors of CHIKV infection that can be blocked by nsP2 and E1 virus proteins. In ZIKV infection, MDA5 and RIG-I expression levels are upregulated in human skin fibroblasts as soon as 6 hpi, with the highest levels at 48 hpi [71]. Moreover, both MDA5 and RIG-I expression levels can be inhibited by NS2A and NS4A of ZIKV in HEK293T cells [217]. Interestingly, Schilling and team announced that only RIG-I functions as a ZIKV detector because the knockout of RIG-I, but not of MDA5, significantly increases virus replication [243]. As 14–3-3ϵ and 14–3-3η proteins promote MDA5 and RIG-I cytosolic-to-mitochondrial translocation, the inhibition of these proteins inhibits downstream RIG-I-like receptor (RLR) signaling. Recently, Riedl and team found that the ZIKV NS3 protein suppresses antiviral signaling via the binding of MDA5 and RIG-I to RLR-14–3-3ϵ/η [244].

Upon the ligand binding of MDA5 and RIG-I, they mediate downstream signal activation through caspase recruitment domain (CARD)–CARD interactions [245]. MDA5 and RIG-I activate mitochondrial antiviral-signaling (MAVS) via its N-terminal CARD that subsequently activates the downstream kinase proteins TBK1 and inhibitor of kappa-B kinase epsilon (IKKε). Vice versa, the protein activates interferon regulator factor (IRF)-3 and nuclear factor kappa B (NF-κB) [246]. The activation of IRF3 or IRF7 and NF-κB induces IFN-β production. Consequently, the triggering of the type I IFN canonical pathway through JAK–STAT signaling results in an ISGF3 complex (STAT1–STAT2–IRF9) assembly that then translocates into the nucleus to activate IFN-stimulated gene (ISG) transcription [247].

Downstream signaling is essential to inhibit both CHIKV and ZIKV infections. However, the viruses have mechanisms to evade IFN responses. Our team documented (using qRT-PCR, PCR array, and Western blot techniques) that the expression level of STAT2 is significantly increased in CHIKV-infected human fibroblasts and markedly decreased in the presence of *Aedes* mosquito saliva [241]. These data imply that *Aedes* saliva affects host cell immune responses to CHIKV infection via, at least, the JAK–STAT-dependent pathway. Recently, Bae and colleagues found that the transfection of CHIKV nsP2, E2 and E1 proteins into HEK293T cells significantly inhibits the downstream signaling of MAVS, TBK1, and IKKε and diminishes NF-κB activation [242]. In addition, a report showed that MAVS knockout-human placental trophoblast cell lines enhance ZIKV infection [219]. NS1 and NS4B overexpression can directly interact with TBK1, preventing TBK1 oligomerization and phosphorylation [216]. 

In another study, ZIKV NS5-HEK293-overexpressed cells directly interacted with IKKε and prevented IRF3 activation [248]. NS5 is also reported to directly bind with endogenous IRF3 and inhibit downstream signaling [61]. Distinct evading mechanisms of ZIKV in the JAK–STAT signaling pathway are characterized by ZIKV NS5 protein–STAT2 protein degradation. The NS5 protein binds and degrades STAT2 in a proteasome-dependent manner [68]. Hertzog and team found that NS5 blocked JAK–STAT signaling via the reduction of STAT2 levels, thus blocking STAT1 phosphorylation [249]. A study also demonstrated that ZIKV NS2B and NS3 inhibited JAK–STAT signaling through the degradation of JAK1 protein in proteasomes, resulting in a reduction of IFN-mediated ISG expression [216].

### 4.3. Updating Host-Targeting Antivirals

Cavinafungin, a linear lipopeptide isolated from *Colispora cavincola* [250], specifically and strongly inhibits ZIKV infection (with IC50 = 0.15 μM and CC50 = 1.65 μM), but it is not effective against CHIKV infection. Using CRISPR/Cas9 gene-editing strategies, it could be determined that cavinafungin targets the host cell’s endoplasmic reticulum-localized signal peptidase (ER-SPase) [251]. Actually, in the ZIKV replication cycle, ER-SPase plays a crucial role in prM/E processing, while the replication of CHIKV mainly occurs in the cytoplasm. This may explain why cavinafungin can be effectively inhibited by ZIKV but not CHIKV. The further study of the function of the ER-SPase in ZIKV polyprotein processing may pave the way for novel ZIKV inhibitor development.

Recently, Prochnow et al. reported that labyrinthopeptins exhibit broad-spectrum antiviral activity mediated by virolysis. Labyrinthopeptins A1 and A2, posttranslational modification peptides isolated from the actinomycete *Actinomadura namibiensis* DSM 6313, are class III lanthipeptides [252,253]. Cell lines pretreated with different concentrations of the compounds before infection by CHIKV and ZIKV inhibit virus infection in the low micromolar-to-nanomolar range. A study of the peptide’s mechanism using 11 species of adsorbed lipids demonstrated that the virolysis effect occurred through the binding of labyrinthopeptins and lipid phosphatidylethanolamine to the viral membrane [254]. However, in vivo experiments of labyrinthopeptins are still needed. 

## 5. Conclusions

In the past few decades, the wide spread of Chikungunya and Zika viruses has highlighted the urgent need for effective antiviral drugs to treat patients and prevent future outbreaks. Several host factors that interact with viral proteins have been identified (Table 1 and Table 2). Nevertheless, the mechanisms of the interplay between virus and host factors during virus infection need further investigation. Such study will expand knowledge and bring more opportunities and ideas for the further development, optimization, and speeding up of the discovery or repurpose of potential therapeutic drugs to counter such re-emerging arboviruses.

## Figures and Tables

**Table 1:** Summary of known host factors that interact with Chikungunya virus proteins. NLS: nuclear localization signal; NES: nuclear exportation signals; E: envelope; nsP: non-structural protein; GAGs: glycosaminoglycans; hTIM: human T cell immunoglobulin mucin; AXL: Axl receptor tyrosine kinase; Mxra8: matrix remodeling-associated protein 8; DC-SIGN: dendritic-cell-specific ICAM-grabbing non-integrin; ER: endoplasmic reticulum.

Viral Protein (Binding Site)	Host Factor/Protein	Host Factor/Protein Function	Host Factor/Protein Involves in Viral Replication	Reference
Capsid (NLS)	Karα4/major binding site	Molecule transportation between nucleus and cytoplasm.	Allow for virus capsid for nuclear translocation.	[16]
Capsid (NES, aa 143–155)	CRM1 (XPO1)/NR	RNA and protein exportation from the nucleus to cytoplasm.	Exit virus capsid from the nucleus.	[16]
E3	Furin	Serine endoprotease with calcium-dependent favor cleaving the paired basic amino acids.	Cleave E3 from pE2-E1 dimer.	[195]
E2	PHB	Various functions, with an especially critical role in proteins and lipids regulating mitochondrial metabolism.	Attach and entry factors.	[40]
E2	GAGs	Cellular process regulation including cell signaling.	Attach and entry factors.	[18]
E2	hTIM1	Human immune response, apoptotic cell engulfment, and T cell proliferation regulation.	Attach and entry factors.	[39]
E2	AXL	Cellular process involvement and regulation.	Attach and entry factors.	[39]
E2	Mxra8	Modulates the activity of various signaling pathways.	Attach and entry factors.	[41]
E2	DC-SIGN	Involved in dendritic cell differentiation, cell adhesion, signaling, migration, and antigen recognition.	Attach and entry factors.	[37]
E2	PTPN2	A tyrosine phosphatase involved in numerous signaling events.	Transport virus structural protein to host cell membrane.	[196]
E2	COL1A2	Type I collagen that strengthens and supports many tissues in the body.	Mechanism unknown.	[196]
E2	ACTG1	Part of cellular trafficking machinery.	Transport virus structural protein to host cell membrane.	[196]
6K/TF	-	-	-	-
E1	COMMD1	Regulation of cellular protein degradation and ubiquitination.	Transport virus structural protein to host cell membrane and regulate host immune responses.	[196]
E1	THBS1	Involved in dentinogenesis and ER stress responses.	Involved in the regulation of host immune responses.	[196]
E1	DYNC1H1	Transfer material such as neurons across cells and important in cell division.	Transport virus structural proteins to host cell membrane and related to neurological manifestation.	[196]
E1	ATP1B3	Sodium/potassium-transporting ATPase.	Fusion factors.	[196]
E1	BST-2	Antiviral response by blocking mature virion budding from host cell.	Budding factors.	[23]
nsP1	BST-2	Antiviral response by blocking mature virion budding from host cell.	Budding factors.	[23]
nsP2	Rpb1	Catalyse RNA transcription.	nsP2 induces Rpb1 degradation, leading to the inhibition of cellular transcription and antiviral responses.	[197]
nsP2	SFRS3/SRp20	Involved in mRNA exportation from the nucleus and RNA splicing.	Mechanism unknown.	[198]
nsP2	CCDC130, CPNE6, POLR2C, MAPK9, EIF4A2, EEF1A1, EIF3I	Putative interactors with nsP2 and mainly involved in apoptosis, transcription, and translation mechanism.	Mechanism unknown.	[199]
nsP2	CEP55, KLC4, TACC3, VIM	Component of cytoskeleton.	Support the formation of replication complex and help to transport in the infected cells.	[198]
nsP2	HNRNPK	Important role in mRNA metabolism, DNA damaging, and activating and controlling the transcription process.	Promotes viral replication.	[198]
nsP2	TTC7B	Regulate and localize phosphatidylinositol 4-kinase to the cell membrane.	Support nsP2 for shutting off the cellular processing of host cells.	[198]
nsP2	ASCC2, EWSR1, IKZF1, TRIM27, ZBTB43, MRFAP1L1(MRG15)	ASCC2: Support gene transcription and repairing. EWSR1: Involved in cell signaling, gene expression. and RNA processing and transport. IKZF1: A transcription factor. TRIM27: Control gene transcription. MRFAP1L1(MRG15): Regulate transcription by the binding with retinoblastoma tumor suppressor (Rb) and MORF4/MRG nuclear protein PAM14. ZBTB43: Suppress Blimp1 transcription process.	Mechanism unknown.	[198]
nsP2	UBQLN4, RCHY1, WWP1	Involved in protein degradation and autophagy.	Promotes viral replication.	[198]
nsP2	GFAP, PDK2, RBM12B, TPR	GFAP: A cell-specific marker helps to differentiate astrocytes from other glial cells. PDK2: Regulate glucose and fatty acid metabolism and homeostasis, cell proliferation, and delay apoptosis. RBM12B: RNA-binding protein. TPR: Support protein and mRNA transportation from the nucleus.	Mechanism unknown.	[198]
nsP2	NDP52/CALCOCO2	Involved in autophagy, inhibit pathogen proliferation.	Support the replication complexes formation.	[198]
nsP3	PI3K-Akt-mTOR pathway	Involved in cellular proliferation and regulate cell cycle.	Support the replication complexes internalization.	[200]
nsP3	G3BP1 and G3BP2	G3BP1: Can be used as stress granule marker and to facilitate stress granule assembly. G3BP2: Could transport mRNA.	Mediate viral replication.	[201]
nsP3	SK2	Involved in cell proliferation, differentiation, and host cell immunity.	Mediate viral replication.	[202]
nsP3	Hsp90β	Maintain cellular homeostasis by modulating cellular processes.	Mechanism unclear.	[203]
nsP4	LCP1	Involved in T cell activation mechanisms.	Mechanism unknown.	[198]
nsP4	Hsp90α	Maintains cellular homeostasis by modulating cellular processes.	Support replication complex formation.	[203]
nsP4	eIF2α	Important for translation process.	Mediate the viral replication.	[204]

**Table 2:** Summary of known host factors which interact with Zika virus proteins. TBK: TANK binding kinase 1; IRF: interferon regulator factor; MAVS: mitochondrial antiviral-signaling; NS: non-structural protein; MTase: methyltransferase; IFN: interferon; MDA5: melanoma differentiation-associated protein 5; RIG-1: retinoic acid-inducible gene-I.

Viral Protein (Binding Site)	Host Factor/Protein	Host Factor/Protein Function	Host Factor/Protein Involves in Viral Replication	Reference
Capsid (Positively charged interface formed by α4 helix)	Nucleotides (single-stranded and double-stranded RNAs or DNAs)	DNA synthesis.	Mechanism unknown.	[205]
Capsid (pre-α1 loop)	Lipid droplets	Not reported.	Virus–host membrane fusion.	[205]
Capsid	G3BP1 and Caprin-1	G3BP1: Essential in innate immune response. Caprin-1: Regulates mRNAs transportation and translation and is involved in neuron synaptic and cell proliferation and migration.	The interaction facilitates viral replication and also impairs stress granule formation.	[206]
Capsid	UPF1	Essential for nonsense-mediated decay (NMD) pathway.	Inhibits the antiviral effect of NMD pathway.	[207]
PrM/M (PrM)	Furin	Serine endoprotease with calcium-dependent favor cleaving the paired amino acids.	Facilitate the viral maturation process.	[208]
E (DII)	Endoplasmic membrane	Synthesis, folding, modification, and transport of proteins.	Membrane fusion.	[55]
E (DIII)	Endosome	Regulate the transportation of proteins and lipids among cellular compartments of the endocytic pathway.	Membrane fusion.	[55]
E	DC-SIGN, HSP70, TIM-1 and TAM receptors (TYRO3, AXL, and MER)	DC-SIGN: dendritic cell differentiation, cell adhesion, signaling, migration, and antigen recognition. TIM-1: regulates human immune response, cell survival, and the clearance of apoptotic cells. HSP70: involved in protein folding and unfolding regulation and protects the cell from oxidative stress. TAM receptors: involved in many cellular processes including cell differentiation, cell survival, migration, and innate immune modulation.	DC-SIGN and TIM-1: involved in viral entry. HSP70: mediate viral entry, replication, and release. TAM receptors: involved in viral entry and innate immune responses modulation.	[71,209,210,211,212,213,214]
E	Mfsd2a	Support blood–brain barrier formation and function.	Impaired brain development	[215]
NS1	TBK1	Regulates inflammatory responses to foreign agents.	Blocks IFN signaling	[216]
NS2A	TBK1	Regulates inflammatory responses to foreign agents.	Blocks IFN signaling.	[61]
	IRF3	Transcriptional regulator of type I IFN-dependent immune responses.	Inhibits the production of type I IFN induced by MDA5/RIG-I signaling pathway.	[217]
NS2B	TBK1	Regulates inflammatory responses to foreign agents	Blocks IFN signaling.	[61,216]
NS2B/3	SEPT2	Involved in actin cytoskeleton organization.	Trigger cell death and stress in hNPC.	[208]
	Jak1	Involved in interleukin-2 and interleukin-10 receptors.	Suppress JAK–STAT signaling.	[216]
NS4A	MAVS	Required for innate immune defense against viruses.	Blocks the IFN signaling.	[218,219]
	IRF3	Transcriptional regulator of type I IFN-dependent immune responses.	Inhibits the production of type I IFN induced by MDA5/RIG-I signaling pathway.	[217]
NS4B	TBK1	Regulates inflammatory responses to foreign agents.	Blockis IFN signaling.	[61,216]
NS5	STAT1	Mediated cellular response to IFNs, cytokines, and growth factors.	Blocks IFN signaling.	[220]
NS5 (MTase domain)	STAT2	Mediated IFN-alpha and IFN-beta signaling.	Blocks IFN signaling.	[68]
